# A case of feline large granular lymphocyte lymphoma with complete remission and long survival by surgical resection and adjuvant nimustine administration

**DOI:** 10.1002/vms3.612

**Published:** 2021-08-17

**Authors:** Makoto Akiyoshi, Masami Akiyoshi

**Affiliations:** ^1^ Akiyoshi Animal Clinic Yamato City Kanagawa Japan

**Keywords:** Chemotherapy, gastrointestinal tract, postoperative management, lymphoma, lymphosarcoma

## Abstract

A 7‐year‐old spayed female Scottish Fold cat presented with a 4‐week history of anorexia, weight loss and vomiting. Abdominal ultrasonography revealed a jejunal mass and a slightly enlarged jejunal lymph node. A fine‐needle aspiration of the mass revealed many round cells with multiple small intracytoplasmic magenta granules. The mass was diagnosed as a large granular lymphocyte (LGL) lymphoma based on cytology. The LGL lymphoma was completely resected via open surgery. The histologic and cytologic evaluations showed no neoplastic findings in the jejunal lymph node, liver, spleen, kidney or bone marrow. The LGL lymphoma was localized to the jejunum. Postoperatively, the cat received chemotherapy with nimustine, l‐asparaginase and prednisolone. The cat is currently receiving nimustine every 6 weeks, without adverse events, and treatment has been administrated a total of 18 times up until day 552. The cat is in a good condition, and the LGL lymphoma has not recurred. Nimustine should be considered one of the effective chemotherapeutic agents in the treatment of feline LGL lymphoma cases in the future.

## Graphical abstract

A case of feline large granular lymphocyte lymphoma with complete resection and nimustine: successful treatment

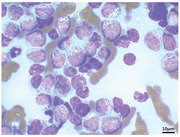

## 1 INTRODUCTION

Felinelarge granular lymphocyte (LGL) lymphoma is a relatively rare subtype of lymphoma, with a poor prognosis and scarce response to chemotherapy (Finotello et al., 2018). Feline LGL lymphoma is not commonly described in the veterinary literature, with only a few articles including large groups of cats available (Finotello et al., 2018; Roccabianca et al., 2006). In the literatures, treatment varied among the diagnosed cats including surgery, chemotherapy such as the CHOP‐based protocol (cyclophosphamide, doxorubicin, vinca alkaloid, prednisolone) and lomustine, radiation therapy, corticosteroids or no treatment (Finotello et al., 2018; Krick *et al*., 2008; Meichner et al., 2014; Moore et al., 2012; Sapierzyński et al., 2015). However, the felines appeared to be minimally responsive to treatment and the median survival time (MST) was just 57 days for treated cats (Finotello et al., 2018). There is limited information describing effective treatment options for feline LGL lymphoma.

Nitrosourea compounds, such as lomustine, have been reported to possess a high antitumour activity in cats with lymphomas and mast cell tumours (Dutelle et al., 2012; Rassnick *et al*., 2008; Rau & Burgess, 2017). Nimustine was developed as a nitrosourea‐derived anticancer agent for humans in Japan and has been reported to have an equivalent or higher cytotoxic activity than lomustine against the murine lymphoid leukaemia (Nagourney et al., 1978). However, there is no report examining efficacy of nimustine in feline LGL lymphoma.

We herein describe a case of feline LGL lymphoma treated by enterectomy and postoperative chemotherapy with nimustine.

## 2 CASE HISTORIES

**FIGURE 1:**
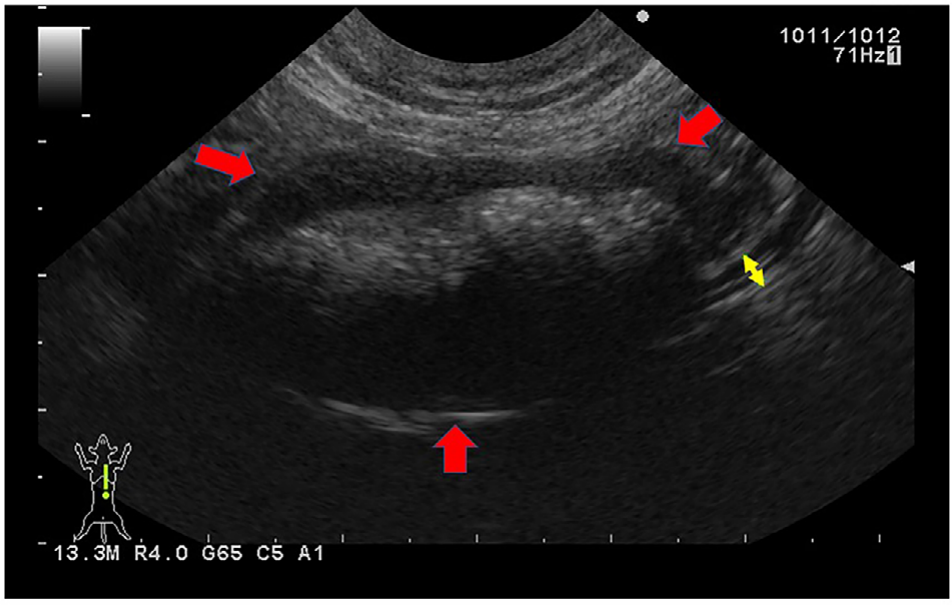
Ultrasonographic findings of the jejunal mass. The red arrows indicate the hypogenic mass in the jejunum. The yellow arrows indicate the normal jejunum wall at the border between the mass and the healthy tissue

**FIGURE 2:**
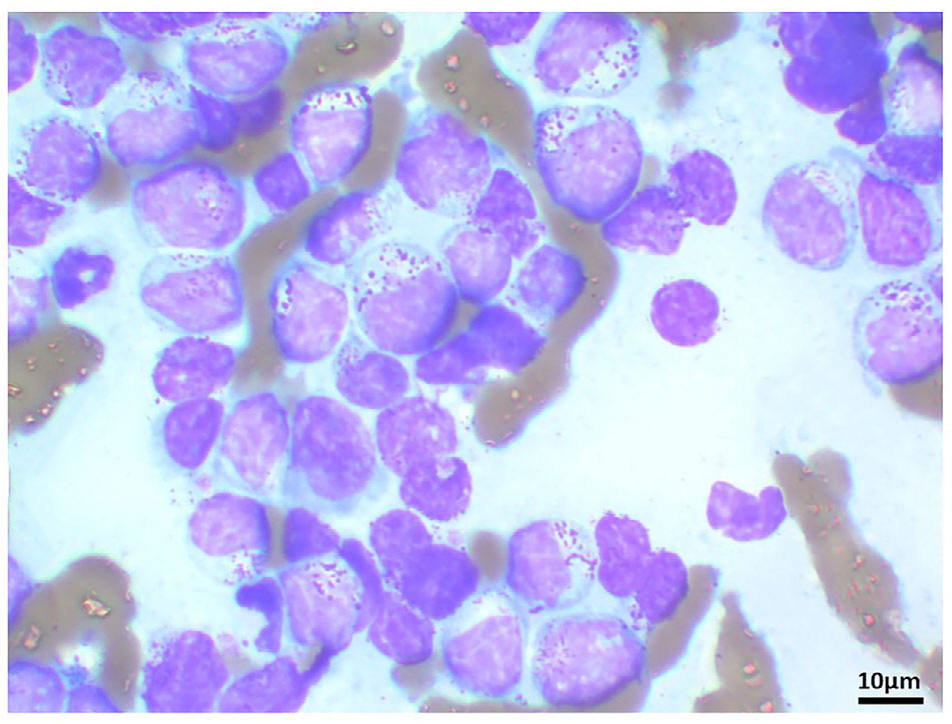
Photomicrographs of the cytologic findings from the jejunal mass. A jejunal mass aspirate showed large granular lymphocytes (LGLs) were present. Wright–Giemsa stain, bar = 10 μm

A 7‐year‐old, 4.5 kg, spayed female Scottish Fold cat was presented with a 4‐week history of anorexia, weight loss and vomiting. Prior to the referral, the cat was administered enrofloxacin (5 mg/kg/day) and maropitant (2 mg/kg/day); however, its clinical signs did not completely resolve. A physical examination revealed a palpable abdominal mass. Complete blood cell count (CBC) revealed no abnormalities (Table 1). Wright–Giemsa‐stained peripheral blood smear revealed no neoplastic cells. Plasma chemistry revealed an increased serum amyloid A (SAA; 45.2 μg/ml, reference interval [RI], < 5.49 μg/ml) (Table 1). Serology tests were negative for the feline leukaemia virus (FeLV) antigen and the feline immunodeficiency virus (FIV) antibody (SNAP, FeLV/FIV Combo; IDEXX Laboratories, Inc). Serum iron was decreased to 39 μg/dl (FUJIFILM VET Systems, Inc; RI: 53–168 μg/dl) (Table 1). No abnormalities were observed upon urine and faecal examination. Thoracic radiographs were unremarkable; however, abdominal radiographs revealed an irregularly rounded, large soft tissue opaque mass (4 cm in diameter) in the caudal ventral abdomen. Abdominal ultrasonography revealed a large irregularly rounded heterogeneously hypoechoic mass (3.5 cm in diameter) connected to the jejunum loop, which had a complete loss of layering, and mostly involved both sides of the wall in the caudal ventral abdomen (Figure 1). The jejunal lymph node was hypoechoic and mildly enlarged (1 cm thick). Peritoneal fluid was not observed. Wright–Giemsa‐stained cytologic mass specimens revealed a proliferation of lymphocytes and that most were intermediate to large in size. The nuclei were round, occasionally indented, paracentral to eccentric with coarse granular chromatin. Rarely, the lymphocytes showed multiple poorly distinct round nucleoli. A moderate amount of basophilic cytoplasm was present, which frequently contained multiple small intracytoplasmic magenta granules. These cytologic findings were suggestive of LGL lymphoma by a clinical pathologist with diplomate of the ACVP (Figure 2).

**FIGURE 3:**
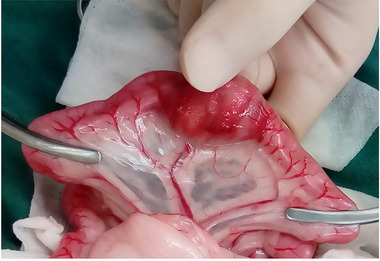
Macroscopic findings from the jejunal mass during open surgery. The mass was localized to the jejunum

On day 1, the cat underwent extirpation of the masses in the jejunum, biopsy of the jejunal lymph node and liver and bone marrow (BM) aspirations. The jejunal mass was surgically removed with a horizontal and border margin of 5 cm from the mass, in addition to a deep margin, as far as the mesentery fat was resectable (Figure 3). In multiple jejunal mass sections, haematoxylin and eosin staining revealed a predominance of neoplastic lymphocytes. The neoplastic lymphocytes were predominantly large with round to oval nuclei, mostly distinct nucleoli with a moderate to abundant amount of eosinophilic to clear cytoplasm. Mitotic figures averaged three per high‐power field. Surgical margins were achieved completely. Immunohistochemical staining was performed for CD3, CD20 and granzyme B using a mouse anti‐CD3 monoclonal antibody (rabbit polyclonal, 1:200; Dako), rabbit anti‐CD20 polyclonal antibody (rabbit polyclonal, 1:400; Thermoscientific), and rabbit anti‐granzyme B polyclonal antibody (rabbit polyclonal, 1:100; Abcam). Immunohistochemical findings from the jejunal mass demonstrated lymphocytes predominantly positive for cluster of differentiation CD3 and granzyme B, with all lymphocytes showing negativity for CD20. In addition, T‐cell receptor gene clonal rearrangements were observed in the jejunal mass using a polymerase chain reaction for antigen receptor rearrangement (PARR) analysis (Kahotechno Co., Ltd.). The jejunal lymph node was reactive without neoplastic changes. The liver also showed no neoplastic changes. Cytologic examinations of the spleen and bilateral kidneys revealed an absence of LGL. According to the findings from the cytologic, histologic immunohistochemical and PARR analyses, the cat was diagnosed with stage I LGL lymphoma localized to the jejunum.

On day 1, prednisolone (subcutaneously [SC], Prednisolone; Zoetis Japan) was initiated at 2 mg/kg/day and cefovecin sodium (SC, Convenia; Zoetis Japan) was administrated at 8 mg/kg. On day 9, the jejunal lymph nodes were of normal size (2.5 mm thick), and recurrence of the jejunal mass indicated that suspected LGL lymphoma was not detected by ultrasonography. In addition, serum SAA concentrations were within the RI, and the clinical signs had improved. On day 9, l‐asparaginase (Leunase; Kyowa Kirin) was administrated at a dose of 400 U/kg SC. In advance, we informed in detail to the owner that nimustine is new in the veterinary field, as well as the expected therapeutic effects, prognosis and side effects of niustine. After that, the owner agreed to use nimustine as an additional chemotherapeutic agent, although there was only one information of its use in cats (Tamura et al., 2013).

**TABLE 1:** Day 0 results of CBC and blood chemistry

		Unit	Reference Interval	Parameters			Unit	Reference Interval	Parameters
RBC	8.73	×106/μL×106/μL	6.54‐12.20	‐	Total proteins	6.3	g/dL	5.0‐7.8	‐
PCV	37.5	%	30.3‐52.3	‐	Albumin	2.6	g/dL	2.6‐4.0	‐
Hemoglobin	13.2	g/dL	9.8‐16.2	‐	ALT	51	IU/L	17‐78	‐
MCV	43	fL	35.9‐53.1	‐	ALP	77	IU/L	47‐254	‐
MCH	15.1	pg	11.8‐17.3	‐	Total Bilirubin	0.1	mg/dL	0.1‐0.8	‐
MCHC	35.2	g/dL	28.1‐35.8	‐	Glucose	131	mg/dL	78‐128	↑↑
Reticulocytes	13.1	×103/μL×103/μL	3‐50	‐	Urea	16.6	mg/dL	10.0‐29.2	‐
Platelets	262	×103/μL×103/μL	151‐600	‐	Creatinine	1.39	mg/dL	0.4‐1.4	‐
					Phosphorous	4.7	mg/dL	1.9‐5.0	‐
WBC	13280	/μL	2870‐17020	‐	Calcium	10.6	mg/dL	9.3‐12.1	‐
Neutrophils	9650	/μL	2300‐10290	‐	T4	2.2	μg/dL	0.9‐3.7	‐
Lymphocytes	2630	/μL	920‐6880	‐	SAA	45.2	μg/mL	<5.49	↑↑
Monocytes	520	/μL	50‐670	‐	Sodium	155	mmol/L	141.0‐152.0	↑↑
Eosinophils	340	/μL	170‐1570	‐	Potassium	4	mmol/L	3.8‐5.0	‐
Basophils	140	/μL	10‐260	‐	Chloride	121	mmol/L	102‐117	↑
					Iron	39	μg/dL	53‐168	↓
PT	9	sec	8.0‐11.0	‐	TIBC	177	μg/dL	211‐458	↓
APTT	12	sec	10.2‐32	‐	UIBC	138	μg/dL	40‐431	‐
Fibrinogen	250	mg/dL	52‐300	‐	EPO	11	mIU/mL	1.9‐22.9	‐
AT	110	%	107‐141	‐	SDMA	9	μg/dL	0‐14	‐
FDPs	2.5	μg/mL	<5	‐	Folate	14.1	μg/L	9.7‐21.6	‐
D‐dimer	0.5	μg/mL	<1.5	‐	Cobalamin	525	μg/L	290‐1000	‐

PCV, packed cell volume; MCV, mean cell volume; MCH, mean corpuscular hemoglobin; MCHC, mean corpuscular hemoglobin concentration; PT, prothrombin time; APTT, activeted partial thromboplastin time; AT, anti‐thrombin; FDPs, fibrin degradation products; EPO, erythropoietin;SDMA, symmetric dimethylarginine; ALT, alanine aminotransferase; ALP, alkaline phosphatase; SAA, serum amyloid A; TIBC, total iron‐binding capacity; UIBC, unsaturated iron‐binding capacity

The effectiveness of nimustine's treatment was evaluated by means of serial ultrasound, and also blood examination including evaluation of peripheral blood smear, and clinical findings. It was decided that these follow‐up examinations should always be performed on the day of nimustine administration and 1 week after nimustine administration, even if the cat was good condition. If ultrasonography revealed abdominal mass suspected to be the recurrence of LGL lymphoma or peripheral blood smear showed the presence of LGL lymphoma, nimustine was considered ineffective and was discontinued. On day 11, a chemotherapy regimen of nimustine (Nidran; Daiichi‐Sankyo) at a dose of 30 mg/m^2^ intravenously, with a decreased prednisolone dosage (1 mg/kg oral administration q24h), was started. However, on day 14, grade 2 anorexia, according to the Veterinary Co‐operative Oncology Group–Common Terminology Criteria for Adverse Events (VCOG–CTCAE), was observed. On day 18, the anorexia had improved with the administration of 2 mg/kg maropitant (Cerenia; Zoetis Japan) once daily, but grade 1 neutropenia (1550/μl, VCOG–CTCAE) was observed (Veterinary Co‐operative Oncology Group, 2016). The neutropenia improved (neutrophil counts, 5800/μl) on day 25. On day 32, no adverse events, including neutropenia, were observed; thus, the second administration of nimustine at a decreased dosage of 25 mg/m^2^ was performed, with 7 days administration of maropitant (2 mg/kg/day). However, on day 39, the adverse event grade 1 anorexia was noticed, which improved with dietary changes, although neutropenia was not observed (neutrophil counts, 5900/μl). Therefore, on day 53, the third dosage of nimustine was decreased to 22.5 mg/m^2^. Afterward, nimustine was continued at a dosage of 22.5 mg/m^2^ every 3 weeks for a total of six times until day 158. Prednisolone was discontinued on day 88 due to the risk of steroid induced diabetes. From day 158, nimustine at a dosage of 22.5 mg/m^2^ with the 7‐day administration of maropitant (2 mg/kg/day) was administered a total of four times every 5 weeks until day 298, and then the cat is receiving nimustine every 6 weeks at the same dosage until day 552, a total of 18 times. Currently, nimustine administration is still on‐going without the change of dosage and interval. The follow‐up examinations revealed the absence of abdominal mass suspected to be the recurrence of LGL lymphoma and the absence of neoplastic cells suspected to be LGL lymphoma infiltration to the peripheral blood. No adverse events were seen except for neutropenia and anorexia after the first administration, and anorexia after the second administration. The cat is currently in a good condition without recurrence of LGL lymphoma or adverse events caused by the nimustine treatment.

## 3 DISCUSSION

Feline LGL lymphoma undergoing an enterectomy only had an MST of 42 days, cats receiving CHOP‐based chemotherapy 60 days and those receiving lomustine 90 days (Finotello et al., 2018; Rau & Burgess, 2017). Therefore, neither surgical treatment nor chemotherapy alone was effective. However, one previous report detailed that a small subset of cats (7.3%, 8 of 109 cats) receiving CHOP‐based chemotherapy (six of eight cats) or undescribed drugs (two of eight cats) survived for more than 6 months, suggesting that a more favourable clinical course can be found for cats with LGL lymphoma (Finotello et al., 2018), although the reason is unclear. There are no studies in the literature that have administered nimustine for the treatment of feline LGL lymphoma, and moreover, there are no studies combining surgery and nimustine.

Nimustine is known to produce an anticancer response via a similar mechanism of action to lomustine (Takahashi et al., 2014; Tamura et al., 2013). However, lomustine is administered orally and nimustine is administered intravenously. Therefore, the dosage of nimustine can be easily tailored and is most suitable for cats that find taking oral medication difficult. Currently, in the veterinary literature, there has been only one report on nimustine for the treatment of feline malignant neoplastic disease (Tamura et al., 2013). In the previous report, nimustine was started at 30 mg/m^2^ IV, every 6 weeks for oligodendroglioma; in total, seven rounds of treatment were administered (Tamura et al., 2013). We believe that this dose was set based on a phase 1 study on nimustine in dogs, and that the administration interval was set to every 6 weeks based on the administration intervals of lomustine in cats (Rassnick et al., 2001; Takahashi et al., 2014; Tamura et al., 2013). Therefore, we also started the first dose at the same dose based on the previous report (Tamura et al., 2013). However, unlike previous reports, the dose interval was every 3 weeks because we wanted to increase drug intensity (Tamura et al., 2013). Our expected toxicity included gastrointestinal symptoms such as vomiting, diarrhoea and loss of appetite, haematological toxicity such as neutropenia and thrombocytopenia, liver damage and renal damage. Adverse events reported were mild and transient leukopenia 1 week post‐nimustine treatment, which recovered within 2 weeks of each administration, and anaphylactic shock after the seventh administration (Tamura et al., 2013). In our case, anaphylactic shock was not observed. In the present report, adverse events were transient neutropenia and anorexia 1‐week post‐nimustine treatment. However, those adverse events improve after 2 weeks and a nimustine dosage of 22.5 mg/m^2^ can be administered 3 weeks later, with no further apparent adverse events observed. Therefore, the nimustine administration interval was set to 3 weeks. Severe adverse events might not develop in cats even if administrating nimustine at a dosage interval of every 3 weeks such as dogs, in contrast to a dosage interval of every 6 weeks on lomustine for cats. Later, the dosing interval was extended to 5‐ and 6‐week intervals, because the follow‐up examinations showed no recurrence, the clinical symptoms were good and the request by the owners with satisfaction of the long‐term prognosis. Additionally, the most concerned reason is that the BM accumulation toxicity by long‐term continuous administration of nimustine should be taken into consideration, but the drug intensity might be better administered for 3 weeks continuously.

There are many limitations to our current report. First, no objective antitumour response has been evaluated due to the absence of measurable LGL lymphoma. The fact that the cat was alive does not necessarily translate into drug activity. Therefore, nimustine's efficiency to LGL lymphoma seen in this report must be interpreted with caution. Second, the number of feline LGL lymphoma cases treated by nimustine is only this cat. Therefore, further investigation will be needed to study the decision of dosage and interval times for nimustine. Third, the cat in our case, not only underwent nimustine treatment, but also a complete surgical resection. The previous study reported that feline gastrointestinal lymphoma with complete surgical margins might survive longer than with incomplete margin or without surgical lymphoma masses resection (Gouldin et al., 2017). We suggest that the complete surgical resection of a LGL lymphoma may also contribute as one of the favourable prognostic factors like this case. In the future, based on the previous case report of nimustine use in a cat (Tamura et al., 2013) and the dose adjustments that needed to be made for the cat in our study, it is required to do a phase I evaluation of nimustine in cats to identify the appropriate dose prior to treating a bunch of cats with LGL lymphoma with nimustine. Moreover, data accumulation of feline LGL lymphomas cases treated by nimustine with and without complete resection is expected.

In conclusion, to the best of our knowledge, this is the first report detailing the successful treatment and long‐term follow‐up of a feline LGL lymphoma and shows that nimustine may be a safe and effective postoperative therapy, and thus this case may present a novel treatment regime for feline LGL lymphoma. A large prospective and randomized evaluation will need to be undertaken in order to determine if nimustine is an appropriate agent for feline LGL lymphoma.

## CONFLICT OF INTEREST

The authors declare no conflict of interest.

### PEER REVIEW

The peer review history for this article is available at https://publons.com/publon/10.1002/vms3.612.
